# The Enactive Approach to Habits: New Concepts for the Cognitive Science of Bad Habits and Addiction

**DOI:** 10.3389/fpsyg.2019.00301

**Published:** 2019-02-26

**Authors:** Susana Ramírez-Vizcaya, Tom Froese

**Affiliations:** ^1^ Philosophy of Science Graduate Program, National Autonomous University of Mexico (UNAM), Mexico City, Mexico; ^2^ Institute for Philosophical Research (IIF), National Autonomous University of Mexico (UNAM), Mexico City, Mexico; ^3^ Institute for Applied Mathematics and Systems Research (IIMAS), National Autonomous University of Mexico (UNAM), Mexico City, Mexico; ^4^ Center for the Sciences of Complexity (C3), UNAM, Mexico City, Mexico

**Keywords:** 4E cognition, dynamical approach, agency, sense-making, regional identities, self, mind-body dualism

## Abstract

Habits are the topic of a venerable history of research that extends back to antiquity, yet they were originally disregarded by the cognitive sciences. They started to become the focus of interdisciplinary research in the 1990s, but since then there has been a stalemate between those who approach habits as a kind of bodily automatism or as a kind of mindful action. This implicit mind-body dualism is ready to be overcome with the rise of interest in embodied, embedded, extended, and enactive (4E) cognition. We review the enactive approach and highlight how it moves beyond the traditional stalemate by integrating both autonomy and sense-making into its theory of agency. It defines a habit as an adaptive, precarious, and self-sustaining network of neural, bodily, and interactive processes that generate dynamical sensorimotor patterns. Habits constitute a central source of normativity for the agent. We identify a potential shortcoming of this enactive account with respect to bad habits, since self-maintenance of a habit would always be intrinsically good. Nevertheless, this is only a problem if, following the mainstream perspective on habits, we treat habits as isolated modules. The enactive approach replaces this atomism with a view of habits as constituting an interdependent whole on whose overall viability the individual habits depend. Accordingly, we propose to define a bad habit as one whose expression, while positive for itself, significantly impairs a person’s well-being by overruling the expression of other situationally relevant habits. We conclude by considering implications of this concept of bad habit for psychological and psychiatric research, particularly with respect to addiction research.

## Introduction

The notion of habit has a long and complex history that can be traced back to Aristotle. Arguably, it is one of the most widely explored notions in the history of Western philosophy and science of mind, occupying a privileged position in the work of prominent figures, such as Hume, Hartley, Hegel, James, Morgan, Bergson, Thorndike, Husserl, Watson, Dewey, Pavlov, Skinner, Merleau-Ponty, Piaget, Hebb, Ricoeur, and Deleuze (Sparrow and Hutchinson, 2013).

Research on habits declined drastically in the mid-1950s with the advent of the cognitive sciences (Barandiaran and Di Paolo, 2014), since by that time this notion had become strongly linked with behaviorism, whose study of habits had focused on finding the laws of association between an external stimuli and an observable response, excluding any reference to mental processes or states (e.g. Watson, 1913; Pavlov, 1927), or even to neural ones (e.g. Skinner, 1938). The cognitive sciences aimed at explaining the cognitive mechanisms and mental processes underlying intelligent behavior, so the study of habits understood as conditioned reflexes was considered irrelevant for their purposes: what behaviorism explained in terms of habits, cognitive science did it in terms of information processing and representations (Barandiaran and Di Paolo, 2014; Wood and Rünger, 2016).

The resurgence of the notion of habit in psychology began within social psychology by the end of the 1990s (e.g. Ouellette and Wood, 1998; Verplanken et al., 1998; Verplanken and Aarts, 1999; Aarts and Dijksterhuis, 2000), drawing mainly from research on automaticity of behavior and dual cognitive processes (e.g. Shiffrin and Schneider, 1977; Hasher and Zacks, 1979; Bargh, 1982; Norman and Shallice, 1986). Neurobiological research on habits also started to proliferate in the 1990s with the discovery of three distinct neural memory and learning systems for explicit or declarative memory, affective memory, and implicit or procedural memory – the latter related to habit learning (e.g. McDonald and White, 1993; Salmon and Butters, 1995; Knowlton et al., 1996; Graybiel, 1998).

Ever since, a growing number of researchers in various branches of psychology and neuroscience have worked on characterizing habits (e.g. Sheeran et al., 2005; Neal et al., 2006), explaining their neurobiological basis (e.g. Graybiel, 2008; Seger and Spiering, 2011; Gremel and Costa, 2013), studying the process of habit formation (e.g. Lally et al., 2010; Kaushal and Rhodes, 2015), developing methods for measuring habit strength (e.g. Gardner, 2015; Labrecque and Wood, 2015; Orbell and Verplanken, 2015), proposing interventions and policies for breaking and creating habitual behaviors (e.g., Verplanken and Wood, 2006; Lally and Gardner, 2013; Rothman et al., 2015), modeling the interaction between habitual and intentional processes in the control of actions (e.g., Daw et al., 2005; Botvinick and Weinstein, 2014; Cooper et al., 2014), and studying the relation of habits with certain psychiatric disorders (e.g., Gillan et al., 2011; Uniacke et al., 2018).

The study of habits has increasingly become an interdisciplinary enterprise, with contributions coming not only from psychology, neurosciences, and philosophy but also from fields such as political science (e.g., Aldrich et al., 2011), organizational studies (e.g., Cohen et al., 2014), marketing (e.g., Ji and Wood, 2007), behavioral economics (e.g. Maréchal, 2010), and transport studies (e.g., Schwanen et al., 2012).

## The Traditional Dichotomist Perspective on Habits

In general, contemporary discussion on habits has been framed by a strong dichotomy between mindfulness and mindlessness that expresses a mind-body dualism: either habits are conceived of as a kind of bodily automatism that opposes to deliberate and intentional actions—which has been the prevalent view in psychology and neurosciences—or as a kind of mindful action. For instance, Wood and Rünger (2016) published a comprehensive review covering a broad spectrum of recent developments in habit research from psychology, neurosciences, and computational modeling. One common conception of habits prevails in the literature reviewed by these authors: habits are regarded as rigid patterns of behavior that are automatically activated by context cues to which they have become mentally associated as a result of having been frequently repeated in the past in a stable context. Accordingly, this conception emphasizes the lack of “awareness, conscious control, cognitive effort, or deliberation” in habit performance (Gardner, 2015, p. 277; for a critical view on the neuroscientific conception of habits, see Bernacer, 2018), which contrasts with the deliberate, conscious, effortful, and goal-directed character of intentional actions. According to Barandiaran and Di Paolo (2014), this notion of habit has its roots in an associationist tradition that dates back to Descartes, Hobbes, Locke, Berkeley, Hume, Hartley, Reid, Bain, Mill, Morgan, and Thorndike, and that influences behaviorism. This trend “conceives of habits atomistically as units that result from the association of ideas or between stimulus and response” (Barandiaran and Di Paolo, 2014, p. 6).

This dichotomist perspective is also present in organization science (Levinthal and Rerup, 2006) and has led to tensions regarding the nature of routines. Drawing mainly from psychological research on procedural memory, some conceptual work has posited organizational routines as habitual (e.g. recurrent interactive patterns, collective habits, or concatenations of individual habits) or somehow based on schemas, scripts, or habits (Cohen, 2012; Turner and Cacciatori, 2016). Given the dominant view of habits in psychology, routines have frequently been conceived as mindless, repetitive, inflexible, and automatically performed, lacking vigilance, explicit deliberation, creativity, and potential for change (e.g. Ashforth and Fried, 1988; Gersick and Hackman, 1990; Cohen, 1991; Louis and Sutton, 1991).

Empirical work has challenged this theoretical understanding of routines by observing that in several organizations routines are “changeable and open to variation” (Becker, 2004, p. 648). In this regard, routines have been defined as having a sequential structure of functionally similar action patterns, whose concrete performance may be highly variable, including a number of exceptions and requiring a considerable deliberation (Pentland and Rueter, 1994). Furthermore, field studies from Feldman (2000) even yield an understanding of routines as “a source of continuous change” (614) due to their work-in-progress character, allowing people to constantly make adjustments and improvements. However, according to this literature, these features of routines (i.e., flexibility and potential for endogenous change) are given by the intentions, reflections, decisions, interpretations, and will of the agents, and not by their having an habitual character, which still seems to be considered a source of inertia and mindlessness (see also Feldman and Pentland, 2003; Levinthal and Rerup, 2006).

Recent proposals have looked for broadening the notion of habit, regarded as a central component of the microfoundation of routines. Following Dewey, Cohen (2007) posits habits as providing “a flexible repertoire of action dispositions that can be customized to some extent as context may require” (p. 781). Turner and Cacciatori (2016) rely on Dewey, Merleau-Ponty, and Bourdieu to propose a typology of habits based on their level of deliberation and variability of context-performance, ranging in a continuum from completely mindless automaticity to more flexible and adaptable ways of responding to changing situations that involve mindfulness and deliberation.

A similar struggle to overcome this mindless-mindfulness dichotomy can be found in contemporary philosophy. In his classic book *The Concept of Mind*, Ryle (1949/2009) makes a distinction between habits and intelligent capacities (i.e., competences and skills). Although for this author neither habits nor intelligent capacities engage propositional content, only the latter involve care, vigilance, judgment, and training —what he calls “knowing how.” On the contrary, “[w]hen we describe someone as doing something by pure or blind habit, we mean that he does it automatically and without having to mind what he is doing” (30). Acting by habit, he claims, is merely a replication of an automatic response to a cue learned by repetition, so it is not a form of know-how. Given this characterization of habits, they have been largely dismissed in analytic philosophy. This is the case even in action theory, despite the ubiquity of habits in everyday life (Douskos, 2017). Furthermore, even authors like Pollard, who give habits a relevant role in the explanation of actions and in the constitution of an agent’s identity, treat them as “patterns of repeated, automatic behavior” that distinguish themselves from “psychological phenomena” (Pollard, 2011, p. 82).

A contrasting position – often called “intellectualist” – has been held by McDowell (2007a,b). According to this author, all human actions are rational in the sense that they are conceptually articulated and guided by reasons. This applies to habits, regarded by him as “embodied coping skills,” which “in mature human beings [are] permeated with mindedness” (McDowell, 2007b, p. 339). McDowell’s view has been strongly opposed from a phenomenological perspective by Dreyfus (2005, 2006, 2007a,b), who claims that “absorbed coping” occurs without attending to the activity being performed (and to an “I” that is the subject who performs it), in a complete absence of mindfulness, only by exercising what Merleau-Ponty calls “bodily intentionality” (Dreyfus, 2007a).

Mainstream theories of habits therefore tend to fall on one or the other side of traditional mind-body dualism. But the complex nature of habits resists being reduced to either side of this dualism: they are too flexible to be mere automatisms, yet they also unfold in a spontaneous manner that does not require constant intentional control. It is therefore no surprise that habits have become an important theme for new approaches to cognitive science that explicitly aim to overcome mind-body dualism.

## Steps Toward a Broader Perspective on Habits

This review focuses on this growing field of research, which has been labeled with the umbrella term of a *4E* (embodied, embedded, extended, and enactive) approach to the cognitive sciences (Rowlands, 2010). This research emphasizes the embodied (Lakoff and Johnson, 1999; Gallagher, 2005; Pfeifer and Bongard, 2007; Chemero, 2009; Shapiro, 2011), embedded (Hutchins, 1995; Clark, 1998; Malafouris, 2013), enactive (Varela et al., 1991; Thompson, 2007; Nöe, 2009; Stewart et al., 2010; Hutto and Myin, 2013; Di Paolo et al., 2017; Gallagher, 2017), and extended (Clark and Chalmers, 1998; Menary, 2010) nature of cognitive processes. Within this broad and diverse collection of perspectives, at least not only four distinct but also intersecting lines of research can be identified.

One has to do with the phenomenological study of habitual body memory (Casey, 2000; Koch et al., 2012; Fuchs, 2016; Proctor, 2016; Tewes, 2018a), which takes the work of Husserl, Bergson, and Merleau-Ponty as its main theoretical background. According to this line of research, habitual body memory is a know-how sedimented in the lived or subjective body that is constantly re-actualized and “gives shape to an individual style of experiencing”, acting, and interacting with the world (Koch et al., 2012, p. 420). This kind of memory, in contrast to episodic memory, “does not ‘presentify’ the past through explicit recollection, but rather reenacts it implicitly” (Fuchs, 2017, p. 335). In this regard, body memory is not considered an inner archive from which one withdraws particular memories, but a dynamical disposition that involves the organism in interaction with its environment. Additionally, as Proctor (2016) points out, habitual body memory can enable spontaneous action and the possibility of transformation, since it “provides enough stability such that one is able to encounter new spaces and experiences with a sense that she knows what she’s doing even though she’s never been there (or done that) before” (256).

A second line of research intersects with the first and is related to the above-mentioned debate between Dreyfus and McDowell on skilled perception and action (Dreyfus, 2005, 2006, 2007a,b; McDowell, 2007a,b). Drawing mainly from phenomenology and psychology, philosophers have started to more systematically explore the relation of habits with attention, conscious awareness and control, intentions, autobiographical remembering, and explicit reasoning (e.g. Sutton, 2007; Sutton et al., 2011; Romdenh-Romluc, 2013; Toner et al., 2015; Cappuccio, 2017; Ingerslev, 2017; Tewes, 2018b). Some of these authors call for a blurring of the strict dichotomy between mind and body that has prevailed in the study of habits. For instance, Sutton (2007) suggests that expert cricket players often employ thinking (e.g. verbal hints and maxims) and episodic memory of previous similar situations to influence their ongoing performance during batting. Similarly, Toner et al. (2015) argue that elite athletes are used to rely on cognitive control (e.g. attention to kinesthetic feedback, bodily self-awareness, dynamic switching of attentional focus, adjustment of movements, and instructional cue words) during skill execution for continuously improving their performance. Others, such as Romdenh-Romluc (2013) and Ingerslev (2017), explore the relationship between habits and agency, challenging the dominant view of action in analytic philosophy (the causal theory of action), which requires actions to be preceded by explicit intentions, to accommodate habits in the realm of actions. Importantly, in contrast to Ryle (1949/2009), this body of literature usually does not make a distinction between skills and habits, but put them in the same category, or regard skills as particular kinds of habits.

A third line of research draws from Material Engagement Theory (MET) (Malafouris, 2013) and the distributed and extended cognition frameworks (Hutchins, 1995; Clark and Chalmers, 1998; Clark, 2008), emphasizing the active role that built environment plays in habitual actions and practical skills. For instance, through historical case studies, Sutton (2008, 2010) argues for an extended notion of memory that goes beyond the individual body to integrate the social and material realm. According to this author, heterogeneous external representational systems or “exograms” play a complementary role in action and cognition by allowing and encouraging “quite different kinds of interaction and coupling” (Sutton, 2008, p. 43). Sutton insists that these material symbols are not disconnected from skill memory, since they can act as nudges for improving the performance of practical skills. Another example of this line of research comes from Ransom (2017). Bringing together MET and an enactive-embodied-dynamical perspective, he argues that what best characterizes the agential process that emerges from the material engagement of an organism with its environment is not the sense of agency, but the phenomenological experience of “*habitual flow* of everyday being in the World” (3), which involves the embodiment of “stable relations within a network of affordances, constraints, and cultural practices of the material culture in which we are situated” (15). In this regard, unlike traditional views on cognition and habits, this line of research does not see the material environment as a mere trigger of processes internal to the individual, but as an essential part of a distributed cognitive system.

Here we will concentrate on a fourth line of research, the enactive approach to habits, because it has the potential to integrate all of these strands into a conceptual framework that is particularly well positioned to overcome the dualism inherent in mainstream research.^1^ Instead of relying on an associationist tradition, the enactive approach that will be reviewed here draws mainly from what Barandiaran and Di Paolo (2014) call an *organicist* tradition in philosophy and psychology, which includes the work on habits of Ravaisson, Bergson, Husserl, Dewey, Heidegger, Goldstein, Merleau-Ponty, Piaget, and Gibson. In general terms, one common feature of the organicist tradition is its view of habits as “a primary ontological phenomenon” (Malabou, 2008, p. vii), shaping the person as a whole and traversing a continuum from the individual to the social, from embodied intentionality to conscious reflection. In this regard, habits are “related to a plastic equilibrium that involves the totality of the organism, including other habits, the body and the habitat they co-determine” (Barandiaran and Di Paolo, 2014, p. 5). Importantly, according to that view, habits are not rigid structures. On the contrary, their plasticity allows for the possibility of change (Ravaisson, 2008).

In the case of the enactive approach, it reassesses this organicist view in the light of dynamical and complex system theories. Taking as a conceptual basis and expanding the theory of autopoiesis by Maturana and Varela (1980), enactivism understands habits as operationally closed, precarious, self-sustaining, and adaptive networks of neural, bodily, and interactive processes that generate dynamical patterns of behavior and constitute a central source of normativity for the agent (Di Paolo, 2005; Barandiaran, 2008; Di Paolo, 2009a,b). Instead of framing the discussion of habits in terms of a contrast between automaticity and mindful actions, this approach invites us to think in terms of a continuity between *biological autonomy* and *sense-making* (Weber and Varela, 2002; Di Paolo et al., 2017). Importantly, enactive research on habits distances itself from the prevalent atomistic perspective in the psychology of habits; it stands out by proposing a topology of mutually dependent habits or regional identities (Di Paolo, 2009a, 2010) that interact in complex ways, enabling and restraining each other (Egbert and Barandiaran, 2014).

In the next section, we will review the enactive approach to habits in more depth. This allows us to uncover an ambiguity in the enactive account that requires further clarification: by focusing on the positive role of self-maintenance in the normativity of habits, it becomes difficult to explain bad habits whose maintenance is undesirable. We sketch a possible response to this ambiguity that emphasizes the interdependent organization of habits into a way of life. We also consider the implications of the resulting enactive concept of bad habit for empirical research, particularly with respect to addictions.

## An Enactive Approach to Habits

Over the last 30 years, work in phenomenology, situated artificial intelligence, autonomous robotics, complexity, and dynamical systems has offered perspectives that emphasize the dynamical, self-organized, embodied, and situated nature of cognition (Newen et al., 2018). One of the approaches that integrates this diverse work into a consistent theoretical framework is enactivism, which sees cognition as “an embodied engagement in which the world is brought forth by the coherent activity of a cogniser in its environment” (Di Paolo, 2009a, p. 12). In the following, we first review the basic concepts of this framework before reviewing its theory of habits in more detail.

### Enactivism: Basic Concepts

One of the central ideas of enactivism is that of *autonomy* (Barandiaran, 2014). According to Di Paolo (2009a), “an *autonomous system* is defined as a system composed of several processes that actively generate and sustain an identity under precarious circumstances” (15). This idea originates in (and expands on) Maturana and Varela’s theory of biological autonomy, according to which *autopoiesis* or self-production is what makes a system a *living* system (Maturana, 1975; Varela, 1979; Maturana and Varela, 1980). This theory regards an autopoietic system as “the minimal living organization” (Weber and Varela, 2002, p. 115), conceived as a network of processes of metabolic production, in which the material components that are constantly being produced sustain that same network that produces them. In this sense, the materiality of an autopoietic system is always changing, while its organization has to remain within certain viability limits.

The idea of being both the outcome and the cause of its constituting processes is expressed in the principle of *operational closure* – also called “organizational closure” by Varela (1979) – which states that the operation of each constituent process of an operationally closed system is conditioned by at least some other process belonging to that same system and, in turn, conditions one or more of those constituent processes. Thus operational closure “arises through the circular concatenation of processes to constitute an interdependent network” (Varela, 1979, p. 55). Operational closure is a condition for autonomy, making it possible for the system to define its own *identity* and distinguish itself from the environment, while remaining open for material and energetic exchange (Thompson, 2007). As Di Paolo (2005) sums it up, “processes of material self-production generate a self-distinguishing concrete unity in the sense that it is sustained by its own activity” (p. 433).

In addition to operational closure, another condition for a system to be autonomous is that of *precariousness* (Weber and Varela, 2002). While operational closure implies that all the processes of an operational closed system are “modulated, adjusted, modified, or coupled to other processes” from that same system, the concept of precariousness adds a further restriction: that the individual processes “also depend for their continuation on the organizational network they sustain”, so that they would eventually extinguish if left in isolation (Di Paolo, 2009a, p. 16). Therefore, the system “as whole is itself the *condition of its parts*” (Fuchs, 2018, p. 85). It is the precariousness of its self-constituted metabolic identity (the potential to die that is intrinsic to life) that is at the origin of autonomous systems having a concern for its own conservation (Froese, 2017).

An autonomous system operates under far-from-equilibrium conditions, so it has to continually strive to counteract its intrinsic entropic tendencies, as well as perturbations from the environment, in order to preserve its metabolic identity. This requires the capacity to regulate its internal and relational states according to the virtual consequences that these entropic trends and external perturbations may have for its conservation. Accordingly, Di Paolo (2005) proposes that an autonomous system must have the property of *adaptivity*, i.e., “the capacity of an organism to regulate itself with respect to the boundaries of its own viability” (430). This property allows such a system to differentiate between encounters with the environment that would otherwise be equally viable, giving it the possibility to avoid potentially “risky situations” and seek for “preferable ones” (Di Paolo, 2009b, p. 50), creating thereby “an intrinsic relation with the world in terms of values and norms, a form of *sense-making*” (Di Paolo, 2010, p. 140). In that way, those situations that contribute to the conservation of its metabolic identity are viewed by the system as “intrinsically *good*,” while those that challenge its subsistence as “intrinsically *bad*” (140). Adaptivity thereby opens a normative dimension grounded on the metabolic organization of the autonomous system (but see Barrett, 2017 for a critical perspective). Moreover, only by being able to regulate its coupling with the environment according to some self-generated norms, an autonomous system can be regarded as a *cognitive agent*, i.e., “a self-constructed unity that engages the world by actively regulating its exchanges with it for adaptive purposes that are meant to serve its continued viability” (Di Paolo, 2005, p. 443).

Nonetheless, survival is not the only value that may guide an agent’s behavior, so, for example, though there may be many equally viable ways of obtaining food, organisms will usually select one behavior over all available options and will persevere in it (Di Paolo, 2010). As Di Paolo et al. (2017) point out, “[s]ome actions are as effective as others are in terms of their biological purpose, but they are preferred because they are habitual and comfortable” (p. 143). This may be explained in terms of an organism’s behaviors getting “increasingly attuned to the regularities of the body and its surrounding so as to achieve what Merleau-Ponty denominates *maximal grip*” (Di Paolo, 2010, p. 146). According to this approach, such repeated behaviors or *habits* conform *habitual identities* or *ways of life* that organisms strive to sustain. One particular illustrative example of this comes from Kohler’s (1964) experiments on visual distortion, “a kind of sensorimotor disruption”, says Di Paolo (2005), “that cannot conceivably be thought of as placing the organism in any *direct* metabolic risk” (p. 446). In this regard, some authors within enactivism (Di Paolo, 2005, 2009a,b, 2010; Barandiaran, 2008; Egbert and Barandiaran, 2014; Di Paolo et al., 2017) have worked on expanding the notion of metabolic autonomy to include habits as autonomous systems that comprehend “partial aspects of the nervous system, physiological and structural systems of the body, and patterns of behaviour and processes in the environment” (Di Paolo, 2009a, p. 18). The complexity of habits can range from very simple movements, such as nail biting or knuckle cracking, to highly complex and dynamic bundles of sensorimotor schemes, such as perceiving and interacting socially all the way to skillfully playing the piano or driving a car. Accordingly, habits open up the world to us, making it familiar and more easily accessible, while at the same time imposing a preferred yet narrower structure on our activities Proctor (2016).

In the next subsections, we delve further into this enactive view on habits and review related research on dynamical systems. This will help to shed light on the empirical study of addictions, as we will show in the final discussion section.

### Habitual Identities and a Topology of Habits

According to enactivism, the main natural norm that guides an agent’s behavior comes from the preservation of its self-constituted metabolic identity: Jonas’ notion of survival as “the mother-value of all values” (Weber and Varela, 2002, p. 111). However, as some authors within enactivism have proposed (Di Paolo, 2005, 2009a,b, 2010; Barandiaran, 2008; Egbert and Barandiaran, 2014; Di Paolo et al., 2017), identity generation can be given at different levels. This results in additional sources of normativity “based on other forms of operationally closed networks of processes, such as socio-linguistic selves, organized bundles of habits, etc.” (Di Paolo, 2009a, p. 19). Hence, non-metabolic values can be rooted in an agent’s habits, organized in an interactional domain as precarious operationally closed networks that underlie the generation of behavior (Di Paolo, 2009b). Thus, habits “work within the boundaries of metabolic viability but are underdetermined by it and can consequently introduce their own normativity” (Di Paolo, 2005, p. 445). In this way, enactivism generalizes the notions of autonomy, autopoiesis, and adaptivity from the biology of the body to the psychology of habits – and potentially, considering the sociocultural constitution of many human habits, to the sociology of habitus.

Along these lines, Egbert and Barandiaran (2014) suggest that the notion of habit “holds the potential to become a blending category between the biological and the psychological” (2). According to these authors, “mental life emerges from a sensorimotor substrata that makes possible the development of an increasingly complex ecology of self-sustaining *sensorimotor* life-forms” (13). Therefore, they suggest that habits may be seen as the most fundamental “building blocks of mental life” (3), in analogy to metabolic autopoietic processes, which are the basic blocks for biological life. For these authors, habits resemble such autopoietic biological processes in that both are “self-maintaining, precarious, dissipative structures that rely upon cyclic processes to persist” and whose “processes of self-maintenance are contingent upon the existence of an appropriate environment” (10).

In order to provide a minimal proof of concept for the analogy between habits and metabolism, two agent-based simulation models using an *itinerant deformable sensorimotor medium* were developed (Egbert and Barandiaran, 2014; Egbert and Cañamero, 2014). These models aim at investigating the formation of habits and the influence of essential physiological variables in the context of diabetes behavioral management. The first model (Egbert and Barandiaran, 2014) shows that self-stabilizing patterns of behavior (i.e., habits) can spontaneously emerge and dynamically adapt through a history of interaction of an embodied agent with its environment (a light source) that generates sensorimotor contingencies, without requiring neither a reward mechanism nor any computations or internal representations of that environment. An important moral of this model is that habits influence the viability of other habits, either preventing them to occur or increasing the chance of its persistence. Accordingly, as Barandiaran (2014) asserts, a sensorimotor agent can be regarded as “an emergent web of habits nested on its behavior generating mechanisms, and the adaptive preservation of the internal stability of this web becomes the normative axis of its ongoing operations” (3). One future avenue for research, according to the authors, comes from exploring the different levels of adaptivity that habits may exhibit, since while some habits may extinguish with mild changes in the environment, some may persist under radical changes by modifying their organization.

The second model (Egbert and Cañamero, 2014) was inspired by the dynamics of diabetes, where hormonal regulation (in this case, insulin and glucagon) is insufficient to keep blood-sugar level within healthy thresholds. It investigates the coupling and mutual stabilization between habits and an essential biological variable (blood-glucose levels) by inducing interoception to sense the state of that variable. Since the essential variable became part of the sensorimotor environment by including it as an interoceptive sensory input, its dynamics were essential for the stability of habits. Consequently, in several of the experimental trials, the only stable patterns of behavior (i.e., habits) that persisted were those that maintained the essential variable within its viability limits because “only reliable interaction with the environment can result in repeated patterns” of behavior (174). Further work is needed to explore the emergence of unhealthy habits that do not maintain the biological essential variables within their viability limits.

Another topic of research is the relationship between different habits. In contrast to the traditional atomistic view on habits, this enactive perspective suggests “a topology of regional identities” (Di Paolo, 2009a, p. 20) coexisting within the same individual (see also Di Paolo, 2005, p. 446) and giving rise to an integrated “life/mind system” or *self* (Di Paolo, 2009a, p. 18). The notion of regional identities is akin to Varela’s (1991, 1999) notion of “microidentities.” The general idea is that agents do not hold monolithic identities that remain constant independently of the activities they perform. On the other hand, this notion does not imply a complete fragmentation of the self, either: it is not that a completely different person will emerge from each interaction, but that particular sets of habits will be regularly displayed by an agent depending on his current activities and context performance. Moreover, regional identities are interrelated and their limits are fluid. Importantly, this new level of identity generation grounds a new level of normativity for agents, since preserving the conditions of viability of their habitual identities becomes a norm that guides their perceptions and actions. In this regard, operational closure and adaptivity can be identified not only in single self-reinforcing habits, but also at the level of an ecology of habits, which “are nested in hierarchical, sequential, and ultimately networked relations in a kind of ecosystem” (Di Paolo et al., 2017, p. 147).

According to Barandiaran (2008), while a single isolated habit would take control of “the behavior generating mechanisms of the agent for its own perpetuation” (282), bundles of habits integrating an autonomous system would self-organize and sustain themselves through their dynamics, establishing a set of viability conditions for the whole system, as well as a milieu of viable interactions that allows it to preserve its overall self-generated identity. By the same token, while a single isolated habit would be executed in a rigid way, an autonomous adaptive network of habits would have a more flexible organization, as long as its identity remains within its viability limits, undergoing “a continuous process of equilibration […] whereby it assimilates new situations, accommodating its organized bundle accordingly” (283). This may require giving up some particular habits for the sake of the whole or adapting the interactions between them to recover stability. Additionally, for agents to be capable of quickly responding to different environmental situations, this stability must be temporary, i.e. the whole network of habits needs to be *metastable*: “They must retain a residue of dynamic criticality without which they would simply be unchangeable automatisms” (Di Paolo et al., 2017, p. 102). In other words, the enactive approach to habits does justice to both their autonomy and their flexibility without privileging one aspect over the other.

### Toward an Enactive Account of Bad Habits

According to the enactive approach, habits are self-sustaining networks of bodily, neural, and interactional processes that become a source of normativity for an agent, in such a way that the preservation of her habitual identities guides much of her perceptions, thoughts, and behaviors. This perspective may seem to make it hard to account for the notion of bad habits, whose normativity seems to be conflicting and difficult to reconcile with other sources of normativity that lead to the agent’s overall well-being. Since habits depend on the viability of the organism, it becomes difficult to conceive of habits that put this viability in danger. However, this problem only emerges if we adopt a traditional atomistic view of habits, regarding them as isolated responses to triggering stimuli. As we saw in the previous section, this atomism is explicitly rejected by enactivism in favor of a more holistic perspective that posits a network of habitual identities as the focus of its research.

In this respect, the notion of “sensorimotor life” has been proposed by Di Paolo et al. (2017) to refer to a form of life constituted by “self-sustaining, habitual organizations” that through their “internal logic, […] constraining relations, and the adaptive facilitation between acts can give rise to a sensorimotor normativity” (7). Accordingly, self-regulation occurs both at the level of a single habit and at the level of an integrated network of habits. Therefore, the activities of an agent are meaningful not only because they contribute to his biological survival, but also because they are conducive “to the stability and coherence of a sensorimotor repertoire” (39). Piaget’s theory of equilibration serves as a relevant framework for them to account for the stabilization of habits through processes of assimilation and accommodation that involve sequences of sensorimotor coordination patterns between the agent and the environment. This continuous process of equilibration includes the transformation, creation, integration, differentiation, elimination, and reorganization of habits.

Importantly, under this enactive account, the self-sustaining habits that constitute an agent may not coexist in perfect harmony, since “the inherent regulative tendencies of sophisticated processes of identity generation are likely to sometimes enter into conflict even with basic metabolic values” (Di Paolo, 2009a, p. 18). This is clearly the case with the so-called bad habits, some of which may take over the global identity and impose their own normativity at the level of the whole network of regional identities, ensuring their preferred enaction even under circumstances that would have normally called for the activation of a different set of habits. In this regard, Di Paolo (2009a) calls attention to the possibility that reliance on a way of life alters the basic autonomy of metabolism to the point of affecting the condition of operational closure of autopoiesis, making it dependent on habits, which incorporate themselves into the agent’s physiology. This may be particularly true for addictive behaviors, such as nicotine addiction, which become so deeply rooted in the individual that they resist most self-control strategies and interventions. However, as argued before, habits can conflict not only with an agent’s metabolic identity, risking her health or even her survival, but also with many of her other regional identities. Accordingly, as Di Paolo (2010) points out, some habits can be bad in the sense that they “may drive the system to situations that are contrary to its own survival or well-being” (148).

Under this approach, our sense of well-being can be understood “as manifestations of the relative coherence of a self-organized form of identity” (Barrett, 2017, p. 434). Let us think of a person—Alice—who is a professional cello player and an amateur mountain climber. These are two regional, socioculturally constituted identities that involve a whole different set of habits and that have to be continuously nourished and negotiated. Besides these identities, Alice is also a vegan activist, has a family life, and enjoys nightlife at clubs with friends. With each new identity, new sources of normativity for guiding behavior are at play. Because of the interrelatedness of regional identities, if one of them is affected, it will have an impact on some of the other identities and thereby even on the whole self. Although Alice may have an adequate balance between her cello player and mountain climber identities, it can be the case that they enter into conflict in particular situations, such as when an orchestra’s rehearsal interferes with an important climbing date or when an injury in her hand after a though climb prevents her from rehearsing. If conflict between regional identities becomes sustained, the agent’s well-being can be put at risk. In this regard, as we argued before, the normativity that guides the agent goes beyond mere biological survival: it also has to do with preserving the relative stability and coherence of the whole set of regional identities.

The notion of regional identities has remained relatively underdeveloped within the enactive approach. However, Varela's (1991, 1999) related notion of micro-identities has been recently brought back to focus by Kiverstein and Rietveld (2018) within the Skilled Intentionality Framework. Drawing from the enactive and the ecological approaches to cognitive science, these authors understand micro-identities as “interrelated states of action-readiness that coordinate to multiple relevant affordances” (154). In this regard, another related way to consider an agent’s well-being is in terms of her relations with the environment, which are expressed in what Rietveld and Kiverstein (2014) call a “field of relevant affordances”. Depending on the particular situation and the regional identities at play, some aspects of the environment become relevant and evoke “bodily states of action readiness” (Rietveld et al., 2018, p. 52) that “reflect a tendency of the individual to modify the relation between herself and the environment in a way that is in line with what matters to her” (55). Thus, under this perspective, the agent’s environment is understood as a “world of value or significance, of affordances having affective allure” (53). As we previously pointed out, agents’ habits involve enabling conditions that are not reduced to the body, but extend to their coupling with the material, social, and cultural environment – including interactions with other people. When enabling conditions in the agent-environment system are in place, the whole network of processes maintains itself (Di Paolo et al., 2017). Otherwise, the agent has to regulate herself and/or her relations with the environment in order to reestablish the metastable patterns of coordination between bodily processes and environmental dynamics that sustain her habits. This regulation can be seen as a movement “toward an optimal grip on multiple relevant affordances simultaneously, that is on a *field of relevant affordances*” (Rietveld et al., 2018, p. 45). In this context, well-being can be understood as an agent’s having a grip on a rich, dynamic, and varied field of relevant affordances, with some solicitations having more relevance (i.e., being more attractive or having a higher affective allure) than others in particular times and situations. Some bad habits (e.g. addictions) can shrink considerably an agent’s field of relevant affordances, thereby reducing “[t]he scope of possibilities for action” (57).

We therefore propose that *a bad habit is a habit whose expression, while positive for its own continued self-maintenance, is negative for a person’s well-being because it consistently overrules the expression of other situationally relevant actions and habits.*^2^ In mild cases, self-control may be able to correct some of this imbalance. But, in extreme cases, the bad habit comes to dominate the whole network of habits that constitutes a person’s way of life, thereby making self-control largely impotent, closing up the agent’s possibilities of transformation, and ultimately even undermining her continued biological viability.^3^

For instance, playing online video games is not intrinsically bad, but it can become a bad habit when it starts to interfere with the performance of other relevant activities, such as doing the house chores, completing homework, socializing with family and friends, etc. As this example shows, a bad habit’s negative valence can be defined in relation to its direct suppression of the activation of other appropriate interaction patterns, and not directly with respect to a negative impact on biological values such as metabolic self-maintenance. Nevertheless, it is possible that a bad habit comes to dominate a person’s way of life to the extent that even biological viability is compromised, as when a person addicted to online interactive video games ends up dying from exhaustion after playing for 2 days straight (Young, 2009).

Note that one habit can be bad for another habit without itself being a bad habit: being a mountain climber can be bad for being a professional cellist if the arm strain of climbing tends to interfere with cello performance. But being a mountain climber only becomes a bad habit, as we defined it, if it is consistently performed at the expense of other, potentially more appropriate activities. Note also that a bad habit’s dominance of the network of habits should not be equated with the rigidity of an automatism: in fact, the realization of a bad habit can be quite creative, as it must often be realized under conditions that are not appropriate or conducive, like trying to smoke a cigarette on a long-haul flight without getting detected or smuggling drugs into a rehab session.

Finally, it is important to remark that our concept of well-being allows for gradations. Accordingly, some gray areas exist in the notion of bad habits developed here that still require to be clarified. For instance, the habit of drinking wine can give a sense of well-being to an agent. This habit may be accompanied by other related habits, such as eating bread and cheese or smoking a cigar while drinking a cup of wine. This network of habits may even get incorporated as an essential part of her social identity. Perhaps they can also constitute a special regional identity if the agent, for example, belongs to a wine tasting community. Even though this identity seems to contribute to the agent’s well-being in the long run, it might pose a severe health problem for her and thereby still end up impairing the enaction of other relevant habits. Further work is needed to better understand this kind of temporally distributed bad habit.

## Discussion

In this final section, we will discuss some implications of the enactive concept of bad habit for empirical research on addictions. In a previous paper (Schütz et al., 2018), we proposed approaching addiction from the enactive notion of habits in order to develop a more comprehensive model that contributes to integrating and making sense of some puzzling phenomena that from a clinical perspective appear to be central to addiction, but that have been largely overlooked by current psychopathology. These aspects include the motivations that lead patients to smuggle drugs into treatment despite their intentions to stop using; the impaired insight into addiction (anosognosia) that prevents patients from truthfully state the amount of daily drug consumption; and the effort that requires switching from supporting others’ consumption to supporting their recovery and abstinence.

In general terms, we proposed that these behaviors could be understood as attempts to maintain “the addict’s form of life, which is being threatened by treatment” (Schütz et al., 2018, p. 4). From this perspective, addiction is not considered in traditional terms of pathological urges that inevitably lead to compulsive drug-seeking and drug-taking. Instead, addiction is seen as “one of the many habitual identities that constitute an addict’s form of life and that is so deeply ingrained into the agent’s physiology that it alters her metabolic autonomy and escalates to dependence” (4). Addiction thus is a *bad* habit in the sense that it jeopardizes or severely restrains the expression of some of the person’s regional identities that are relevant for her overall well-being, such as the biological and social ones. In this regard, we can say that in addiction one regional identity takes control over the global identity. Philosophers such as Dewey and Deleuze had previously acknowledged the relation between habits and self. Dewey (1922), for instance, considered that a habit has a power over us “because it is so intimately a part of ourselves. It has a hold upon us because we are the habit. […] All habits are demands for a certain kinds of activity; and they constitute the self” (24–25). Thus, the normativity that guides behavior is centered on maintaining this addictive identity, making it utterly difficult for the agent to exert self-control.

Addiction also reduces the richness of relevant affordances that is usually available to an agent. In addiction, an agent is open and responsive to just a few possibilities for action related to her addiction identity, losing sight of other possibilities related to regional identities that once they cared about, such as those linked to hobbies, job, or family life. For instance, when drug addiction dominates an agent’s identity, a nightclub that once solicited dancing, singing, and meeting new people, now becomes just a place to buy drugs; many everyday objects, such as roach clips, spoons, mirrors, straws, or cards, now become drug-related paraphernalia that evoke states of bodily action readiness for the agent. At the same time, the relevance of those places, objects, persons, and activities that once moved an agent gradually dissolves until the micro-world of addiction comes to dominate her field of relevant affordances. In this regard, as Proctor (2016) states, “addictions are ways of being in the world that can be distinguished from other habits […] in terms of the difficulty of their disruption combined with the increasingly world-narrowing consequences” (259). Using dynamical systems terms, we can claim that “addicts are stuck in a suboptimal attractor, which creates a tension that may manifest as frustration or anxiety for not being able to develop other regional identities” (Schütz et al., 2018, p. 4). This tension, in turn, can lead agents to relapse in an attempt to improve their affective situation, understood as a relative increase of grip on a restricted way of life. However, by doing so, agents move to a sub-optimal grip on their wider field of affordances (Rietveld et al., 2018), given the shrinkage of possibilities for action and the silencing of other regional identities resulting from addiction, as well as the affective tension that results from the recurrent efforts to overcome it.

We argue that this ecologically enriched enactive systems perspective on habits may hold clues for how to better treat severe cases of addiction. What seems to be needed is a way of effecting a holistic reorganization of the addicts’ way of life, in order to allow them to overcome the attractive pull of their sub-optimal grip. In particular, among the various treatments and interventions that have been designed and implemented for addiction, psychedelic therapy combined with psychological support has shown some promise (Carhart-Harris and Goodwin, 2017; Kyzar et al., 2017). For instance, a long-term follow-up study on smoking cessation (Johnson et al., 2016) found that the administration of psilocybin (two to three moderate to high doses) together with cognitive behavioral therapy (CBT) had high success rates, with 67% of the participants biologically confirmed as smoking abstinent at 12-month follow-up, and 60% at even longer-term follow-ups (an average of 30 months post-treatment). Another study (Bogenschutz et al., 2015) also provides evidence of significant clinical improvement in alcohol dependence (percent of heavy drinking and drinking days significantly lower than baseline) following the administration of psilocybin in combination with a psychosocial intervention, while a meta-analysis of randomized controlled clinical trials (Krebs and Johansen, 2012) found a significant beneficial effect of single high or very high doses of lysergic acid diethylamide (LSD) on alcoholism in the short-term (2–3 months post-treatment) and medium-term (6 months post-treatment). However, the mechanism of efficacy of psychedelic treatments for addictions still remains poorly understood.

According to Carhart-Harris et al. (2014), psychedelic drugs may cause a disruption in the regular pattern of neural activation that is the basis of the sense of self. Psychedelic drugs have been found to increase entropic brain activity, including disruption of resting-state functional connectivity (Carhart-Harris et al., 2014; Kyzar et al., 2017). In particular, “psychedelics may induce a brain state whereby established resting state networks break down, and novel local connectivity hubs form between regions that show little connectivity in a baseline state” (Kyzar et al., 2017, 1,000). This neurobiological effect of psychedelics can be approached from an enactive perspective as a way to allow addicted persons to escape a *sub-optimal attractor configuration* of the network of habits constituting their way of life. More specifically, we propose that this “reset” may lead them to attain more global optima that, at least in part, manage to integrate the regional habitual identities into a more coherent self by excluding bad habits. This may also allow agents to acquire a broader perspective in relation to the possibilities that had remained closed to them because of addiction, thereby broadening their field of relevant affordances.

A model by Woodward et al. (2015) supports this idea. These authors implemented a self-optimizing spiking neural network model based on ideas developed by Watson et al. (2011). The process consists in a three-step iterative routine that starts with (1) a random initialization of the neural activity, (2) allowing it to converge to an attractor, which represents a viable solution to a constraint satisfaction problem, and it ends with (3) the application of a small amount of Hebbian learning, so that the network forms an associative memory of the different attractors it has visited. The basic finding is that this associative memory enlarges the basins of attraction of global optima, even if they have not been visited yet, making them easier to find. Woodward et al. (2015) suggest that the iterative alteration of normal neural activity is a fundamental element of the self-optimization process, since “the more each ‘reset’ deviates from previously visited state configurations, the more likely it is that the neural network will converge on a novel attractor, and thereby implicitly learn more about the layout of its own overall state space” (17). In this regard, the authors also hypothesize that ritual practices that temporarily alter the state of consciousness of its participants may act as such a “global neural reset” with similar therapeutic benefits (18).

Interestingly, a case study performed with an Amazonian community of religious ayahuasca users within the Santo Daime movement who have a ritual attendance of about six times per month shows significantly lower scores in the Medical Status, Alcohol Use, and Psychiatric Status subscales of the Addiction Severity Index (ASI) compared to a non-ayahuasca using Amazonian community. Although it remains to be seen whether this finding can be generalized to other communities, it suggests that the neural self-optimization model is on the right track. Moreover, given that the operational conditions of self-optimization might also be realized at the level of social networks in terms of communal patterns of interaction including its ritualized interruptions (Froese, 2018), it is tempting to speculate that this kind of community is also healthier as a social whole. In other words, the next step for the enactive approach may be to continue the expansion of the concept of habit from biological autonomy to psychological habits to sociocultural habitus.

## Author Contributions

SR-V wrote the initial draft of the manuscript after discussion with TF. Both authors revised the manuscript several times and agreed upon the final version.

### Conflict of Interest Statement

The authors declare that the research was conducted in the absence of any commercial or financial relationships that could be construed as a potential conflict of interest.

## Footnotes

^1^A distinction has been made between three kinds of enactivism: sensorimotor, radical, and autopoietic-adaptive (Hutto and Myin, 2017). In this review, we will concentrate on the autopoietic-adaptive enactivism, which is more often simply known as the “enactive approach” (Di Paolo and Thompson, 2014).

^2^We acknowledge that this is just one out of many ways for a habit to be bad. A different account of bad habits has been provided by Bernacer and Murillo (2014). These authors consider cognitive control as the key factor for distinguishing good and bad habits, arguing that cognitive control allows the agent to “direct his or her behavior more adequately to the goal.” Accordingly, they distinguish a good habit (habit-as-learning) as that whose acquisition “implies a better cognitive control of the actions related to that habit” and a bad one (habit-as-routine) as a habit that “involves rigidity and blurs the goal” (5). Comparing this view with the one presented here is an exciting possibility for future work.

^3^We see biological health as an important constituent of well-being.
